# Diagnostic and predictive power of plasma proteins in Alzheimer's disease: a cross-sectional and longitudinal study in China

**DOI:** 10.1038/s41598-024-66195-7

**Published:** 2024-07-30

**Authors:** Wei Li, Lin Sun, Ling Yue, Shifu Xiao

**Affiliations:** 1 Department of Geriatric Psychiatry, Shanghai Mental Health Center, Shanghai Jiao Tong University School of Medicine, Shanghai, 200030 China; 2 Alzheimer’s Disease and Related Disorders Center, Shanghai Jiao Tong University, Shanghai, China

**Keywords:** Alzheimer disease, Plasma proteins, Simoa, Globus pallidus, NfL, Psychology, Biomarkers

## Abstract

Convenient and effective biomarkers are essential for the early diagnosis and treatment of Alzheimer’s disease (AD). In the cross-sectional study, 103 patients with AD, 82 patients with aMCI and 508 normal controls (NC) were enrolled. The single‐molecule array (Simoa) technique was used to assess the levels of plasma proteins, including NfL, T-tau, P-tau-181, Aβ40, Aβ42. Montreal Cognitive Assessment (MoCA) was used to assess the overall cognitive function of all subjects. Moreover, Amyloid PET and structural head MRI were also performed in a subset of the population. In the follow-up, the previous 508 normal older adults were followed up for two years, then COX regression analysis was used to investigate the association between baseline plasma proteins and future cognitive outcomes. NfL, T-tau, P-tau-181, Aβ40, Aβ42 and Aβ42/40 were altered in AD dementia, and NfL, Aβ42 and Aβ42/40 significantly outperformed all plasma proteins in differentiating AD dementia from NC, while NfL and Aβ42/40 could effectively distinguish between aMCI and NC. However, only plasma NfL was associated with future cognitive decline, and it was negatively correlated with MoCA (r = − 0.298, p < 0.001) and the volume of the left globus pallidus (r = − 0.278, p = 0.033). Plasma NfL can help distinguish between cognitively normal and cognitively impaired individuals (MCI/dementia) at the syndrome level. However, since we have not introduced other biomarkers for AD, such as PET CT or cerebrospinal fluid, and have not verified in other neurodegenerative diseases, whether plasma NFL can be used as a biomarker for AD needs to be further studied and explored.

## Introduction

Alzheimer's disease (AD) is a major cause of dementia and one of the major challenges facing health care in the twenty-first century^1^. In general, the symptoms of disease begin with mild memory impairment and develop dysfunctions in complex daily activities, and several other aspects of cognitive function. When AD is clinically diagnosed, neuropathologic lesions and neuronal loss occur in many brain regions^2^. Early and accurate diagnosis of AD can greatly improve the prognosis of patients^3^, however, the current AD can only be definitively diagnosed post mortem.

Biomarkers that detect changes in amyloid B (Ab) peptide and Tau protein in vivo can improve the diagnosis and detection in subjects at risk for AD^4^. According to the guideline of the National Institute on Aging and Alzheimer's Association in 2011, the biomarkers for AD can be classified into three categories: β amyloid deposition (Aβ), pathologic tau, and neurodegeneration [AT(N)]^5^. Imaging-to-autopsy comparison studies have determined that amyloid positron emission tomography (PET) is an effective in vivo substitute for deposits (in brain parenchyma/vascular walls)^6,7^, while CSF Aβ42 or the Aβ42/ Aβ40 ratio is also a valid indicator of the abnormal pathologic state associated with cerebral Aβ^8^. What’s more, the introduction of PET ligands for pathologic tau is also an important development^9,10^.

However, although these above biomarkers can reflect the pathological process of AD, lumbar puncture is an invasive technigue, and amyloid-PET is very expensive and not a screen tool^11^. This highlights the need for less invasive and inexpensive biomarkers that can predict central amyloid status. Since blood is more readily available than CSF, there is no doubt that blood sampling is more suitable than CSF sampling for the detection of AD biomarkers, whether for clinical diagnosis or screening, or for repeated sampling in clinical trials^12^. Plasma Aβ42 appears to be a potential plasma protein, but it was only mildly associated with amyloid pathology in the brain^13^. Other plasma proteins, such as Tau protein, phospho-tau181(ptau 181), and neurofilament light (NfL) chain, have been shown to be helpful in the diagnosis of AD, but the results have been inconsistent^14^.

Developing a blood biomarker for AD is difficult for several reasons: first, brain-derived biomarkers usually have relatively low concentrations in the blood because the blood–brain barrier prevents molecules from freely traveling between the central nervous system and blood compartments^15^.Second, platelets and other extracerebral tissues can also secrete Aβ proteins^16^. Third, the minute amounts of brain proteins entering the bloodstream must be measured in a matrix containing high levels of plasma proteins such as IgG and albumin, which introduces a high risk of interference into the analytical method^17^; Fourth, in addition to dilution, brain proteins released into blood may be degraded by proteases, cleared by the kidneys or metabolized in the liver^18^; Fifth, plasma biomarker detection also has its limitations due to the lack of consensus on the boundary value, which can not be used as a basis for diagnosis, but only as an important means of stratified screening; Further, there may be heterophilic antibodies in blood but not in CNS, which may give falsely low or high results^15^. Nevertheless, technical developments in the field of mass spectrometry and ultrasensitive immunoassays have given new hopes^19^.

The single‐molecule array (Simoa) technique, which is based on immunocapture of the protein biomarker on magnetic beads, is trapped in femtolitre volume wells, followed by the addition of enzyme‐labelled detection antibody and digital quantification, allowing precise quantification of trace protein to subpicogram levels per mL (quantification limit: 0.04 pg ml^−1^)^12^. This new analytical techniques has shown 25‐and 126‐fold higher sensitivity than the electrochemiluminescence(ECL) and enzyme‐linked immunosorbent assay (ELISA), respectively^20^. In the current study, we used Simoa to detect five AD related plasma proteins, including Aβ (Aβ42 and Aβ40), tau(P-tau-181), neurodegeneration [total-tau (T-tau) and NfL]. By detecting these proteins, especially in the earlier stages of AD, we can better understand the pathological process of AD, and hopefully develop new, non-invasive biomarkers for AD.

## Methods

### Study populations

Data were obtained from a cohort study on the health of the elderly in Shanghai (http://www.shanghaibrainagingstudy.org/). This project was launched in 2016 and was a prospective observational cohort study. This project aimed to understand the mortality, prevalence, incidence, and population distribution characteristics of mild cognitive impairment (MCI) and Alzheimer’s disease (AD) among elderly individuals aged > 55 years in Shanghai communities. A total of 82 aMCI patients, 103 AD patients and 508 normal controls (NC) were included in the current study. Among them, the aMCI and NC patients were from the community, while the AD patients were from Shanghai Mental Health Center. At the baseline stage, all the subjects completed the clinical diagnosis, neuropsychological test and detection of plasma proteins. Subsequently, we followed up the normal elderly for 2 years (n = 506), the process was the same as the baseline, but no plasma proteins were detected at this time.

#### Diagnostic criteria for the normal elderly

Subjects would be considered normal elderly if they (1) Age 55 or above; (2) scored 26–30 points on the Montreal Cognitive Assessment (MoCA) at the screening visit^21^; (3) without cognitive symptoms as diagnosed by a physician; (4) without visual or hearing impairment; (5) did not meet the diagnosis of mild cognitive impairment (MCI) or dementia. The exclusion criteria were (1) presence of an acute illness or serious mental illness (e.g. myocardial infarction, stroke, acute infection, delirium, major depression, and schizophrenia); (2) refuse to collect plasma; (3) Misuse of alcohol or substances.

#### Diagnostic criteria for mild cognitive impairment due to Alzheimer's disease (aMCI)

The diagnosis of aMCI was based on the recommendations from the national institute on aging-Alzheimer’s association workgroups on diagnostic guidelines for Alzheimer’s disease^22^: (1) concern regarding a change in cognition; (2) impairment in one or more cognitive domains; (3) preservation of independence in functional abilities; (4) not demented. In addition, the diagnosis of aMCI also requires the support of magnetic resonance data, such as obvious atrophy of the hippocampus.

#### Diagnostic criteria for Alzheimer's disease (AD)

The AD patients were assessed by a medical doctor specialized in dementia disorders. All the participants with AD met the DSM-IIIR criteria for dementia as well as the NINCDS-ADRDA criteria for AD^23^. In addition, patients with AD should meet either a positive of amyloid PET scans or a positive of Aβ 42 protein in CNS. The exclusion criteria were 1) vascular dementia, frontotemporal dementia, and Lewy body or Parkinson dementia; 2) major depression according to geriatric depression scale (GDS 20/30) or DSM IV; other diseases that might interfere with cognitive evaluation.

Ethical approval was obtained from the Ethics Committee of the Shanghai Mental Health Center, Ethical number: 2018-11C1, and all participants or their legal guardians signed informed consent prior to the study. The whole study was carried out in accordance with the principles of the Declaration of Helsinki.

### Plasma sampling and the Simoa analysis

All the participants’ plasma samples were collected at 7–9 am after an overnight fast. Plasma tubes were centrifuged at 1800 × *g* for 10 min and different tubes of plasma from different individuals were brought together in order to achieve a high volume. The different plasma pools are placed in a polypropylene tube (Sarstedt, Germany) and stored at − 80 °C until use. Hemolysis status should be checked prior to analysis; if hemolysis occurs during sample collection or processing steps it may require discarding the sample or adjusting interpretation of results accordingly. The analysis process of Simoa is as follows: EDTA plasma Aβ40, Aβ42, Tau, pTau181 and NfL were quantified using an ultra-sensitive Simoa technology (Quanterix, MA, US) on the automated Simoa HD-X platform (GBIO, Hangzhou, China) per manufacturer’s instruction. The multiplex Neurology 3-Plex A (Cat. No. 101995), NF-light (Cat No: 103186) and pTau181 V2 (Cat. No. 103714) assay kits were purchased from Quanterix and used accordingly. Plasma samples were diluted at a 1:4 ratio for all assays. Calibrators and quality controls were measured in duplicate. All sample measurement was performed on a single run basis. The assays were performed using kits with the same lot number. Operators were unaware of participants’ disease status.

#### Limitations and improvement methods of simoa technology

At present, although Simoa technology has been successfully commercialized, with the advantages of automation and high throughput, its application is still mainly focused on basic laboratory research, and its clinical application is very limited. The clinical application of Simoa technology is limited mainly by the following factors: First, the trace markers that have been found are still limited, and their clinical significance has not been fully clarified; Secondly, compared with the existing chemiluminescence detection, Simoa single molecule immunoassay takes a longer time and is a batch detection, which cannot realize the on-call detection of specimens. Third, single-molecule immunoassay instruments and consumables are more expensive, which limits their promotion and use to a certain extent. Therefore, discovering more trace markers and clarifying their clinical significance, and developing a more efficient and mature single molecule immune detection platform based on low-cost, easy to operate and POCT detection microdroplet technology will help promote the clinical application of single molecule immune detection technology, thus providing a new means for the diagnosis and prognosis of diseases.

### T1 structural magnetic resonance

T1-Brain structure image (including 63 NC) was acquired by using a Siemens Magnetom Verio 3.0 T scanner (Siemens, Munich, Germany). The parameters of T1-weighted 3D magnetization prepared rapid gradient echo (MPRAGE) sequences were as follows: TR = 2,300 ms, TE = 2.98 ms, matrix size = 240 × 256; flip angle of 9 degrees, field of view (FOV) = 240 × 256 mm; slice thickness = 1.2 mm. Volumetric data was assessed by automated procedures, which have been described by wolz R^24^ et al. For each subject, volume and asymmetry with various brain areas as well as cortical thickness were extracted directly using FreeSurfer v6.0.

### Amyloid PET

A subset of participants (including 9 NC, 19 aMCI patients, and 12 AD patients) received amyloid PET scans of [18F] Florbetaben (target dose 8.1 mCi). The standard uptake ratio (SUVR) was calculated using data from 50 to 70 min after injection and data from the subcerebellar gray matter reference area in natural space. FreeSurfer was used to segment the brain and obtain region of interest (ROI) from T1-weighted MRI. Based on the ROI defined by Freesurfer, the integrated SUVR is the average of the SUVR of the frontal, temporal and parietal cortex. SUVR had a cut-off score of 1.25, which was considered as amyloid positivity.

### Cognitive function

Global cognition was assessed using the Beijing version of the Montreal Cognitive Assessment (MoCA-BJ), which is a brief screening test for cognitive impairment that covers major cognitive domains including attention, memory, language, orientation, visuospatial ability, and executive functions^25^. Previous studies have shown that MoCA-BJ can effectively distinguish between normal controls, MCI, and dementia among the Chinese elderly with various age and levels of education^26,27^. In the current study, MOCA-BJ was used to assess cognitive function at baseline and follow-up.

## Statistical analysis

Demographic, clinical, and plasma proteins findings were presented as mean (standard deviation) or frequencies (%). Demographic and clinical characteristics were assessed using Fisher exact test across the whole group, and then Kruskal‐Wallis test was used to compare continuous variables between two groups (NC, aMCI, and AD). Means levels of plasma proteins were calculated in the three groups and compared with generalized linear regression with adjusted for age, gender, education level, hypertension, diabetes, and hyperlipidemia. Receiver‐operating characteristic curve (ROC) was used to explore the accuracy of plasma proteins in the diagnosis of AD. Partial correlation analysis (diabetes, hypertension, and hyperlipidemia were controlled) was used to explore the association between plasma proteins, MoCA and cranial magnetic resonance. Cox regression analysis was used to explore the association between baseline plasma proteins and future cognitive changes, with diagnosis as the dependent variable and time to transition as the time variable. Statistical analysis was performed using the SPSS 22.0 software. In all analyses, the two-sided a-level of 0.05 was used for significance testing.

## Results

### Demographical, Daily habits and clinical characteristics (baseline)

693 participants (82aMCI, 508NC and 103 AD) were included in the current study. The average length of education of the normal control group was higher than that of the aMCI group and the AD group (p < 0.05), while there was no statistical difference (p > 0.05) between the aMCI group and the AD group. Hypertension, diabetes, hyperlipidemia differed (p < 0.05) between groups, while no difference (p > 0.05) was found in age, gender, CHD, cerebral hemorrhage, stroke and depression between groups. Table 1 presents the results.

**Table 1:** Comparison of general demographic data and clinical data among three groups (cross-sectional).

Characteristics	Whole sample (n = 693)	NC (n = 508)	aMCI (n = 82)	AD (n = 103)	p value
Age, years (SD)	69.36 (7.56)	69.22 (7.20)	69.68 (7.65)	69.96 (9.54)	0.664
Education, years (SD)	10.83 (3.64)	11.39 (3.37)*^,&^	8.65 (3.74)	9.63 (4.01)	< 0.001
Male, n (%)	253 (37.2)	183 (36.0)	27 (32.9)	43 (47.8)	0.072
Hypertension, n (%)	372 (54.8)	290 (57.1)	50 (61.0)	32 (36.0)	0.001
Diabetes, n (%)	161 (23.7)	126 (24.8)	24 (29.3)	11 (12.4)	0.018
CHD, n (%)	94 (13.8)	68 (13.4)	17 (20.7)	9 (10.1)	0.111
Hyperlipidemia, n (%)	250 (36.8)	201 (39.6)	31 (37.8)	18 (20.2)	0.002
Cerebral hemorrhage, n (%)	14 (2.1)	9 (1.8)	1 (1.2)	4 (4.5)	0.221
Stroke, n (%)	109 (16.1)	79 (15.6)	17 (20.7)	13 (14.6)	0.457
Depression, n (%)	14 (2.1)	10 (2.0)	2 (2.4)	2 (2.2)	0.954
NfL, pg/ml, mean (SD)	21.33 (22.21)	17.52 (11.19)*^&^	20.35 (12.67)^&^	40.92 (46.22)	< 0.001
Ptau181, pg/ml, mean (SD)	3.10 (2.93)	2.79 (2.08)^&^	3.04 (2.66)^&^	4.65 (5.31)	< 0.001
Ttau, pg/ml, mean (SD)	2.60 (2.81)	2.45 (2.38)*^&^	2.90 (1.75)	3.10 (4.74)	0.063
Aβ42, pg/ml, mean (SD)	11.07 (3.19)	11.36 (3.04)*^&^	10.70 (3.79)	9.96 (3.12)	0.001
Aβ40,pg/ml, mean (SD)	192.61 (64.84)	175.09 (45.07)*^,&^	255.93 (74.09)^&^	228.62 (89.46)	< 0.001
Aβ42/ 40, mean (SD)	0.06 (0.02)	0.07 (0.02)*^,&^	0.04 (0.01)^&^	0.05 (0.01)	< 0.001
Baseline MoCA, mean (SD)	22.32 (5.77)	24.52 (2.89)*^,&^	17.95 (3.49)^&^	8.56 (5.73)	< 0.001

*NC* Normal controls, *aMCI* amnestic mild cognitive impairment, *AD* Alzheimer's disease, *CHD* Coronary heart disease, *NfL* neurofilament light, *P‐tau181* phosphorylated tau 181, *T‐tau* total tau, *Aβ42* amyloid‐β42, *Aβ40* amyloid‐β40, *Aβ42/40* amyloid‐β42/40, *MoCA* Montreal Cognitive Assessment.

Data were presented as mean (standard deviation) or frequencies (%); Demographic and clinical characteristics were assessed using Fisher exact test across the whole group, and then Kruskal‐Wallis test was used to compare continuous variables between two groups (NC, aMCI, and AD). Significant differences were found in plasma biomarker concentrations after accounting for the effects of age, educational, sex, hypertension, diabetes, and hyperlipidemia.

*Significantly different from aMCI, p < 0.05.

^&^Significantly different from AD, p < 0.05.

### Plasma proteins across different diagnostic groups (baseline)

**Figure 1:**
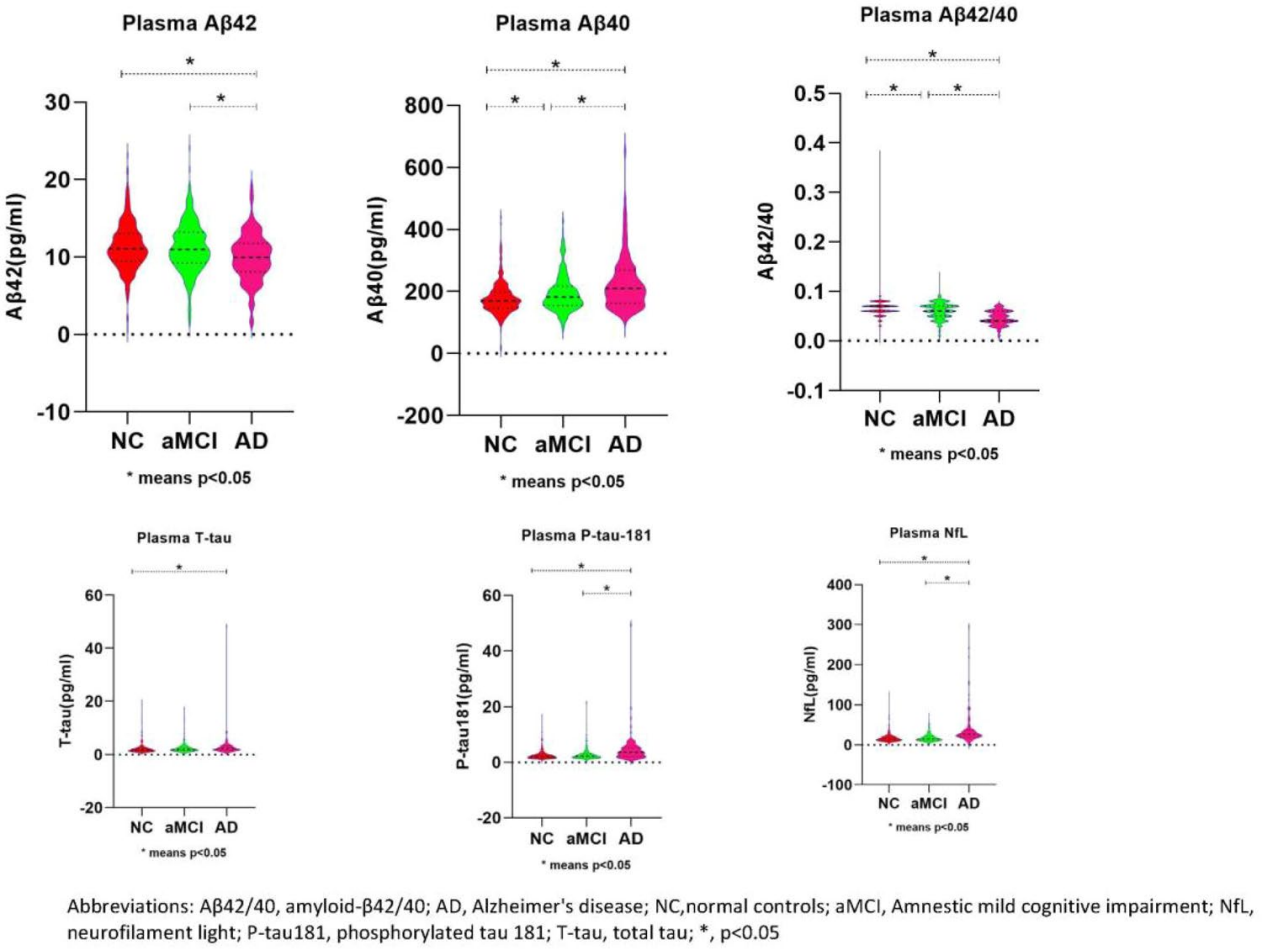
Figure 1 Comparisons of plasma biomarker concentrations across different groups. This figure illustrates the differences in the expression of plasma proteins: Aββ42, Aββ40, Aββ42/40, T-tau, p-tau-181, and NFL in different cognitive states (NC, aMCI, and AD). *Aβ42/40* amyloid-β42/40, *AD* Alzheimer's disease, *Nc* normal controls, *aMCI* amnestic mid cognitive impairment, *NfL* neurofilaments light, *P-tau181* phosphorylated tau 181, *T-tau* total tau. *p<0.05.

By using the generalized linear regression with adjusted for education level, hypertension, diabetes, and hyperlipidemia, we found that the levels of plasma NfL,T-Tau, Aβ40 in normal controls were lower than that in aMCI patients, while Aβ42 and the ratio of Aβ42 to Aβ40 was higher than that in the aMCI group (p < 0.05). However, there was no statistical difference (p > 0.05) in Ptau181 between the two groups. Increased levels of NfL, P-tau181, T-tau, Aβ40 and decreased Aβ42, Aβ42/40 were found in AD patients compared with normal controls (p < 0.05). Moreover, increased levels of NfL, Ptau181, Aβ40, Aβ42/40 and decreased Aβ40 were also found in AD patients compared with aMCI patients (p < 0.05), while there was no statistical difference (p > 0.05) in T-tau protein and Aβ42 between the two groups. (Table 1 and Fig. 1).

### Comparative diagnostic performance of different plasma proteins (baseline)

**Figure 2:**
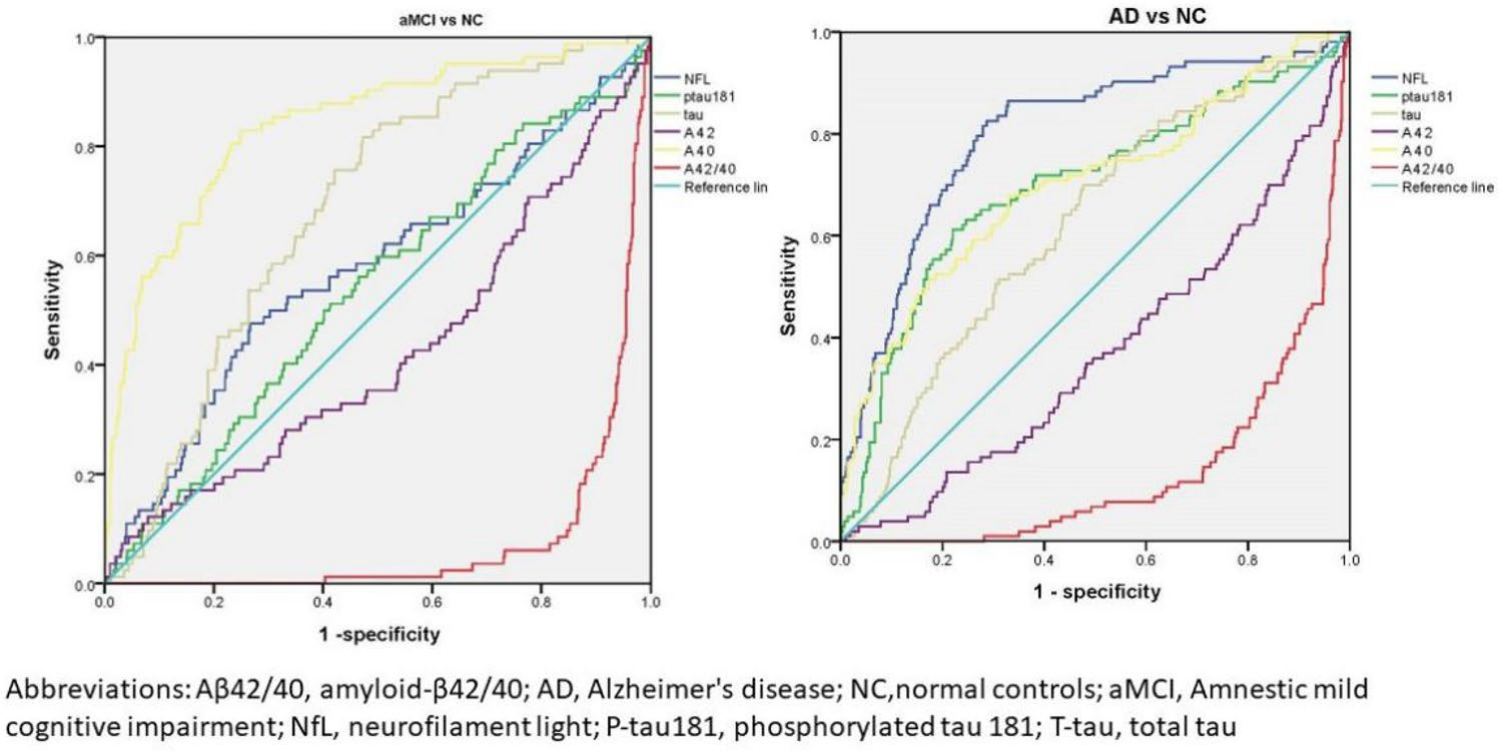
ROC curve was used to compare the accuracy of plasma proteins in the diagnosis of aMCI and AD. This figure shows the predictive power of different plasma proteins (Aββ42, Aββ40, Aββ42/40, T-tau, p-tau-181, and NFL) for aMCI and AD. *Aβ42/40* amyloid-β42/40, *AD* Alzheimer's disease; *Nc* normal controls, *aMCI* amnestic mid cognitive impairment, *NfL* neurofilaments light, *P-tau181* phosphorylated tau 181, *T-tau* total tau.

Receiver‐operating characteristic curve (ROC) was used to explore the accuracy of plasma proteins in the diagnosis of aMCI and AD. For aMCI, the areas under the curve were arranged in descending order as Aβ40 (0.558), T-tau (0.532), NfL(0.478), Aβ42(0.473) and Aβ42/40(0.471); For AD, the areas under the curve were arranged in descending order as NfL(0.791), Ptau181(0.693), Aβ40 (0.653), T-tau (0.599), Aβ42(0.383) and Aβ42/40 (0.197). By using logistic regression analysis, NfL and Aβ42/40were confirmed to be correlated with aMCI, while NfL, Aβ42 and Aβ42/40 (p < 0.001) were confirmed to be associated with AD. Table 2 and Fig. 2 presents the results.

**Table 2:** Relationship between baseline plasma proteins and baseline AD and aMCI.

Characteristics	B	S.E	Wald	df	p	OR	95% confidence interval
aMCI							
Education	− 0.195	0.043	20.387	1	< 0.001*	0.823	0.756–0.896
NfL	− 0.045	0.019	5.764	1	0.016	0.956	0.921–0.992
Ptau181	0.049	0.083	0.346	1	0.556	1.050	0.893–1.235
Aβ42	0.165	0.191	0.749	1	0.387	1.179	0.812–1.714
Aβ40	0.011	0.009	1.433	1	0.231	1.011	0.993–1.029
Aβ42/40	− 149.031	40.748	13.376	1	< 0.001*	–	–
AD							
Education	− 0.142	0.040	12.760	1	< 0.001^&^	0.867	0.082–0.938
NfL	0.049	0.012	18.057	1	< 0.001^&^	1.050	1.027–1.075
Ptau181	0.078	0.060	1.706	1	0.192	1.081	0.962–1.216
Aβ42	− 0.284	0.113	6.293	1	0.012^&^	0.753	0. 603–0.940
Aβ40	0.012	0.007	3.163	1	0.075	1.012	0.999–1.026
Aβ42/40	− 45.534	20.701	4.838	1	0.028^&^	0.763	0.415–0.538

*NC* Normal controls, *aMCI* amnestic mild cognitive impairment, *AD* Alzheimer's disease, *CHD* Coronary heart disease, *NfL* neurofilament light, *P‐tau181* phosphorylated tau 181, *T‐tau* total tau, *Aβ42* amyloid‐β42, *Aβ40* amyloid‐β40, *Aβ42/40* amyloid‐β42/40, *MoCA* Montreal Cognitive Assessment.

Data were presented as mean (standard deviation) or frequencies (%); Demographic and clinical characteristics were assessed using Fisher exact test across the whole group, and then Kruskal‐Wallis test was used to compare continuous variables between two groups (NC, aMCI, and AD). Significant differences were found in plasma biomarker concentrations after accounting for the effects of age, educational, sex, hypertension, diabetes, and hyperlipidemia.

*Significantly different from aMCI, p < 0.05.

^&^Significantly different from AD, p < 0.05.

### Association of plasma proteins with cognitive score and clinical features (baseline)

The results of correlation analysis showed that increased age was associated with NfL, P-tau181, Aβ42, Aβ40, and Aβ42/40, but not T-tau; years of education was associated with NfL, Aβ40, and Aβ42/40, but not P-tau181, T-tau, and Aβ42; gender was only associated with NfL, but not P-tau181, T-tau, Aβ42, Aβ40 and Aβ42/40; MoCA was associated with NfL, P-tau181, Aβ40 and Aβ42/40, but not T-tau, and Aβ42; partial correlation analysis (age, education and gender were controlled) showed that MoCA was still associated with NfL, P-tau181, Aβ40 and Aβ42/40, but not T-tau, and Aβ42.

### Demographical, Daily habits and clinical characteristics (follow up)

After 2 years of follow-up, of the 506 elderly people with normal cognitive function, 370 remained normal (NC-NC), but 136 converted to aMCI (NC-aMCI). The average age of the NC-NC group was lower than that of the NC-aMCI group, while the average length of education was higher than that of the NC-aMCI group (p < 0.05). No difference (p > 0.05) was found in gender, hypertension, diabetes, hyperlipidemia, CHD, cerebral hemorrhage, stroke and depression between the two groups. By using the generalized linear regression with adjusted for age and education level, we found that the levels of plasma NfL, P-tau181, Aβ42 and Aβ40 in NC-NC groups were lower than that in NC-aMCI patients, while there was no statistical difference (p > 0.05) in T-tau and Aβ42/40 between the two groups (Table 3).

**Table 3:** General demographic data of subjects in longitudinal follow-up.

Characteristics	Whole sample (n = 506)	NC-aMCI (n = 136)	NC-NC (n = 370)	p value
Age, years (SD)	69.23 (7.22)	71.26 (6.81)	68.48 (7.23)	0.009*
Education, years (SD)	11.39 (3.37)	10.67 (3.97)	11.66 (3.08)	0.050
Male, n(%)	182 (36.0)	56 (41.2)	126 (34.1)	0.145
Hypertension, n (%)	289 (57.1)	77 (56.6)	212 (57.3)	0.919
Diabetes, n (%)	126 (24.9)	40 (29.4)	86 (23.2)	0.165
CHD, n (%)	68 (13.4)	23 (16.9)	45 (12.2)	0.186
Hyperlipidemia, n (%)	200 (39.5)	55 (40.4)	145 (39.2)	0.838
Cerebral hemorrhage, n (%)	9 (1.8)	2 (1.5)	7 (1.9)	1.000
Stroke, n (%)	79 (15.6)	21 (15.4)	58 (15.7)	1.000
Depression, n (%)	10 (2.0)	2 (1.5)	8 (2.2)	1.000
NfL, pg/ml, mean (SD)	17.54 (11.21)	20.25 (12.46)	16.54 (10.55)	0.001*
Ptau181, pg/ml, mean (SD)	2.79 (2.08)	3.07 (2.52)	2.69 (1.89)	0.033*
T-tau, pg/ml, mean (SD)	2.46 (2.39)	2.46 (2.52)	2.45 (2.34)	0.665
Aβ42, pg/ml, mean (SD)	11.36 (3.05)	11.85 (3.18)	11.18 (2.99)	0.024*
Aβ40,pg/ml, mean (SD)	174.97 (45.12)	181.81 (47.59)	172.45 (43.98)	0.027*
Aβ42/ 40, mean (SD)	0.07 (0.02)	0.07 (0.03)	0.07 (0.02)	0.907
Baseline MoCA, mean (SD)	24.52 (2.89)	23.54 (3.24)	24.88 (2.67)	0.002*
Follow up MoCA, mean (SD)	23.51 (3.73)	19.93 (3.36)	24.82 (2.91)	< 0.001*

*NC* Normal controls, *aMCI* amnestic mild cognitive impairment, *AD* Alzheimer's disease, *CHD* Coronary heart disease, *NfL* neurofilament light, *P‐tau181* phosphorylated tau 181, *T‐tau* total tau, *Aβ42* amyloid‐β42, *Aβ40* amyloid‐β40, *Aβ42/40* amyloid‐β42/40, *MoCA* Montreal Cognitive Assessment.

Data were presented as mean (standard deviation) or frequencies (%); Demographic and clinical characteristics were assessed using Fisher exact test across the whole group, and then Kruskal‐Wallis test was used to compare continuous variables between two groups (NC, aMCI, and AD). Significant differences were found in plasma biomarker concentrations after accounting for the effects of age, educational, sex, hypertension, diabetes, and hyperlipidemia.

*Significantly different from aMCI, p < 0.05.

### Association between baseline plasma proteins and future cognitive outcomes (follow up)

By using Cox regression analysis (LR forward), with diagnosis as the dependent variable and the time to transition as the time variable, we found that baseline plasma NfL was associated with future cognitive decline (p = 0.005, 95% confidence interval:1.005–1.026). Table 4 presents the results.

**Table 4:** Association between baseline plasma proteins and cognitive status at follow-up.

Variables	B	S.E	Wald	df	p	95% confidence interval
NfL	0.015	0.005	8.031	1	0.005*	1.005–1.026

*NfL* neurofilament light.

### Association of plasma proteins with structural magnetic resonance (follow up)

After adjusting for age, sex, education, hippocampus, amygdala and diagnosis, NfL was only found to be associated with the volume of left Globus Pallidus (r = -0.278, p = 0.033), but not right Globus Pallidus (r = 0.052, p = 0.684). However, the results of the linear regression mediation model (MoCA total scores were considered as dependent variables, while NfL and volume of the left globus Pallidus were independent variables) showed that NfL did not affect the cognitive total score through the volume of the left pallidum.

## Discussion

In the current study, we applied the Simoa technique to explore the expression of six plasma proteins (NfL, Ptau181, T-tau, Aβ42, Aβ40 and Aβ42/40) in the plasma of AD patients, aMCI patients and NC elderly, and followed up these NC participants for two years, and finally found that 1) among the six plasma proteins, NFL, Aβ42 and Aβ42/40 could effectively distinguish AD from normal elderly, while NFL and Aβ42/40 could effectively distinguish aMCI from normal elderly; 2) only baseline plasma NfL was associated with future cognitive decline; 3) NfL was negatively correlated with MOCA total score and volume of left globus pallidus, but there was no obvious mediating effect among the three factors.

Neurofilament light chain (NfL) plays an important role in axon transmission and function maintenance, and is the most abundant intermediate filament protein in myelinated subcortical axons^28^. Previous studies pointed that NFL is an ideal marker of large-caliber axonal degeneration, and increased NfL levels incerebrospinal fluid(CSF) are like to reflect neurodegeneration-related axonal injury^29^, such as Alzheimer's disease (AD), frontotemporal lobar degeneration (FTLD), multiple sclerosis and amyotrophic lateral sclerosis^30,31^. In addition, CSF NfL levels have also been proved to be an effective way to distinguish FTLD from AD^32,33^. However, NfL in cerebrospinal fluid is not applicable and difficult to accept for many elderly people, Therefore, blood-based measurement of NfL might be more desirable, as the collection of blood samples is relatively less invasive and more applicable^34^.

So far, there have been a few studies on NfL expression patterns in blood of AD patients. For example, In Lewczuk P’s study, they found that the plasma NfL concentration in patients with Alzheimer's disease was higher than that in the uncorrected control group^35^. In Niklas Mattsson’ study, they found that plasma NfL was associated with AD diagnosis and with biochemical, imaging and cognitive hallmarks of the disease^36^. Liu S^37^ et al. found that gastric cancer subjects expressed lower plasma NfL levels but AD subjects expressed higher plasma NfL levels than normal controls. Hu H^38^ et al. found that plasma NfL concentration and its rate of change had already increased abnormally in the preclinical phase of AD. And Lin YS^39^ et al. also found that plasma NfL was significantly increased in the AD group, compared with the control, mild cognitive impairment (MCI), non-demented Parkinson's disease (PD), and Parkinson's disease dementia groups. In our current study, we found that NfL could effectively distinguish normal from AD or aMCI patients, and help predict the future cognitive status of the elderly with normal cognitive function. These conclusions suggest NfL in plasma may represent a biomarker of cognitive decline in AD, and it is possible to mark the onset of neurodegeneration in subjects at risk for AD familial disease^28^. However, the mechanism of NfL regulates cognition is not very clear.

Neuroimaging using Magnetic Resonance Imaging (MRI) has been widely used to describe the atrophy pattern of cognitive related brain regions in AD and FTLD as well as to find differential trajectories along the different stages of the disease^40–42^. Structural MRI of medial temporal atrophy (MTA) is considered to be a biomarker for an early diagnosis of MCI and AD^43,44^, specifically speaking, volume reduction of medial temporal lobe, including amygdala, and hippocampus has been proved to be an early manifestation of AD^45^. The globus pallidus (GP), a major component of the basal ganglia (BG), communicates with a wide range of cortical regions and supports a variety of functions, including cognition, motivation, and action^46^. Previous studies suggest that deep brain stimulation (DBS) of the globus pallidus internus can effectively improve motor and cognitive symptoms of Parkinson's disease (PD)^47,48^. For patients with Alzheimer's disease, susceptibility in the globus pallidus (GP), may also be developed as a new biomarker for cognitive decline, amyloid deposition, and off-target binding of the tau ligand^49^. Therefore, we focus on the above three brain regions (hippocampus, amygdala, globus pallidus) as our regions of interest.

The relationships between classical AD biochemical markers AD and neuroimaging features and their reciprocal influence have been studied during both the preclinical phases and clinical of the disease. For example, Mattsson N^36^ et al. pointed out that high plasma NfL levels in the MCI and AD cohorts were associated with smaller hippocampal volumes, thinner cortices and larger ventricular. Bhan A found that cerebrospinal fluid (CSF)-NfL at baseline was significantly associated with the rate of atrophy in hippocampus and globus pallidus as evaluated by MRI^50^. In our study, we did not find an association between NfL and the hippocampus and amygdala, but found it to be negatively correlated with the left globus pallidus. Therefore, our conclusions are partially consistent, and the reasons for the differences may be related to race, different disease types and different sources of NFL.

In addition to NFL, we also used Simoa technique to detect other plasma proteins such as Aβ42, Aβ40, T-tau and P-tau181. Although some plasma proteins, such as P-tau181, Aβ42, and Aβ40, differed between groups, these differences disappeared after regression analysis, suggesting that they were not sensitive to the detection of AD. In Fubin Jiao’s study^51^, they found that the multifactor model of plasma Aβ42 and t-tau in combination with MoCA could be a viable model separate health and AD subjects in clinical practice. In Suzanne E Schindler’s study, they found that plasma Aβ42/Aβ40 could be used to screen for individuals likely to be amyloid PET-positive and at risk for AD^52^. Kai-Yuan Tzen^53^ et al. found that only Aβ42/40, not tau, could provide an indirect estimation of Aβ deposition in the brain. In Tao Wang’s study, they used An enzyme-linked immunosorbent assay used to analyze the concentration of the following blood plasma proteins: Aβ42, Aβ40, IL-10, IL-6, phosphorylated tau 181, and total tau, and found that only plasma Aβ40 was able to distinguish between AD and NC groups^54^. A meta-analysis of 83 studies showed that Aβ42, Aβ42/40, NfL and p-tau-181could effectively discriminate AD patients from controls, but it needs to rely on the novel platforms, such as Simoa and immunomagnetic reduction (IMR)^55^. Therefore, the results are inconsistent or even contrary.

We have to admit that our study has some limitations: first, there was no appropriate biological marker to enroll the normal elderly and aMCI patients, and there may be some errors in the division of the two groups; second, we only followed up normal elderly people for two years, and the short follow-up time was also a major limitation of this study; third, the relationship between NfL and globus pallidus needs to be further explored in animal studies; Four, differences in educational attainment, hypertension, diabetes, and hyperlipidemia in the cohort (the NC group compared to the other groups) may have contributed to the findings. Five, Plasma NFL has not been compared with any established independent biomarkers of potential AD pathology (e.g. CSF AD biomarker, PET AD biomarker) and is also a limitation of the current study; Finally, since the current study did not include other diseases, including cerebral infarction, cerebral hemorrhage, etc., whether NFL can be used as a biomarker for AD still needs to be further studied and explored.Finally, only 12 of the 103 AD patients met the conditions for PET CT or cerebrospinal fluid AB42 protein positivity, which is a major limitation of the study.

## Conclusions

Plasma NFL can effectively distinguish between normal elderly and AD or aMCI patients, and the mechanismmay be related to structural changes in the globus pallidus. However, since we have not introduced other biomarkers for AD, such as PET CT or cerebrospinal fluid, and have not verified in other neurodegenerative diseases, whether plasma NFL can be used as a biomarker for AD needs to be further studied and explored.

## Data Availability

The datasets used and/or analyzed during the current study are available from the corresponding author on reasonable request.
